# Hypoglycemic and hypolipidemic activities of phlorizin from *Lithocarpus polystachyus* Rehd in diabetes rats

**DOI:** 10.1002/fsn3.2165

**Published:** 2021-02-06

**Authors:** Wei Zhang, Shiqi Chen, Hualin Fu, Gang Shu, Huaqiao Tang, Xiaoling Zhao, Yang Chen, Xiangyue Huang, Ling Zhao, Lizi Yin, Cheng Lv, Juchun Lin

**Affiliations:** ^1^ Department of Pharmacy College of Veterinary Medicine Sichuan Agricultural University Chengdu China

**Keywords:** diabetes, hyperglycemia, hyperlipidemia, *Lithocarpus polystachyus* Rehd., phlorizin, SD rats

## Abstract

The objective of this study was to investigate the hypoglycemic and hypolipidemic effects of phlorizin on sweet tea in rats with diabetes. Diabetic rat model was established by feeding with HFD (high‐fat diet) and then treating with intraperitoneal injection of STZ (streptozocin). The experiments were divided into therapeutic and preventive experiments. In both experiments, rats were divided into normal, diabetic control, positive control, and phlorizin groups. Symptoms of diabetes, fasting blood glucose (FBG) levels, serum lipid parameters, and pathological changes in the pancreas and liver were evaluated. It was found that the symptoms of diabetes were improved by phlorizin treatment. In addition, phlorizin could decrease FBG, improve serum lipid levels, protect against damaged pancreas islet, and decrease fat deposition in hepatic cells. These effects of phlorizin can be shown only attain to a certain dosage. It can be concluded that phlorizin has the therapeutic and preventive effects on hyperlipidemia and hyperglycemia in diabetes rats.

## Graphical abstract

This study has confirmed that phlorizin from sweet tea has the therapeutic and preventive effects on hyperlipidemia and hyperglycemia in diabetes rats, and phlorizin can be used as a natural health drink to prevent diabetes.

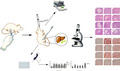

## 1 INTRODUCTION

*Lithocarpus polystachyus* Rehd, also known as sweet tea (ST), has been used as a health drink for thousand years in Chinese folk (Zhou et al., 2013). According to the traditional application, ST has special advantages including antihyperglycemia, antihypertension, antihyperlipidemia, and diabetes improvement. Some other bioactivities of ST, antioxidant, anti‐inflammatory, antianaphylaxis, and hepatoprotective activities, have also been demonstrated by modern pharmacological studies (Li et al., 2005).

It was reported that the main active ingredients of ST are flavonoids, that is, phlorizin, quercetin, and dihydrochalcone (Sheng‐Hua et al., 2010). Among them, phlorizin, a natural product and dietary ingredient first isolated from the bark of the apple trees in 1835 (Petersen, 1835), has been used to improve diabetes in the majority of studies (Choi, 2016; Wang et al., 2016). Literatures have indicated phlorizin, as the specific and competitive inhibitor of SGLT1/2, is able to reduce blood glucose by downregulating intestinal and the renal glucose absorption, restore normal blood glucose levels and normalize insulin sensitivity, and improve dyslipidemia and diabetic complications in diabetic rodent models (Katsuda et al., 2015). In order to further identify the key roles of phlorizin on prevention and therapeutic of diabetes, in this paper we focus on the hypoglycemic and hypolipidemic effects of ST phlorizin orally in high‐fat diet (HFD)/STZ‐induced diabetic rats. It is the first report on the study of preventive effects of phlorizin in diabetes.

## 2 MATERIALS AND METHODS

Phlorizin was isolated from leaves of ST, as previously described (Chen et al., 2017). SD rats (180–220 g) were purchased from Chengdu Dossy Experimental Animals Co., Ltd. All experimental procedures involving animals were approved by the Sichuan Agricultural University Animal Care and Use Committee (registration No. SCAU2018092301). The rats were treated in accordance with Guide for the Care and Use of Laboratory Animals (8th edition, National Academies Press). They were fed standard food pellets and acclimatized in the laboratory animal room for 1 week. Diabetic model rats were fed with HFD (Guo et al., 2002) (food ratio: standard food 58.5%, refine lard 10%, cholesterol 1%, cholate 0.5% and yolk powder 10%, saccharose 20%) for 4 weeks, and then treated with intraperitoneal injection of freshly prepared STZ (40 mg/kg) dissolved in 0.1 M of citrate buffer (pH 4.5). Three days after injection, fasting blood glucose level (FBG) of rats was detected by Roche glucometer. Rats with FBG ≥ 11.1 mmol/L were considered to be hyperglycemia (Wang et al., 2016).

In therapeutic experiment, diabetic rats were randomly divided into six groups (*n* = 10): normal control group (NC), diabetic control group (DC), phlorizin group treated with 30 mg/kg phlorizin (low dose, LD), 60 mg/kg phlorizin (middle dose, MD), and 120 mg/kg phlorizin (high dose, HD), and positive control group treated with 100 mg/kg melbine (DMBG). Phlorizin and melbine were administrated to the rats by ig once daily for 28 days. During the therapeutic period, diabetic symptoms such as behavioral changes and metabolic index (water consumption, food consumption, urine outputs) were recorded every day. The body weight, FGB, and serum levels of cholesterol (TC), triglyceride (TG), low‐density lipoprotein cholesterol (LDL‐C), and high‐density lipoprotein cholesterol (HDL‐C) were determined weekly throughout the study period.

In preventive experiment, healthy rats were randomly split into six groups (*n* = 10). Except NC, fed with standard food, other groups, including phlorizin prevention groups (LD: 30 mg/kg, MD: 60 mg/kg, HD: 120 mg/kg), ginsenoside group (GS; 80 mg/kg), and DC were fed with HFD. Phlorizin and ginsenoside were orally administrated to rats fed with HFD once daily for 28 days. On the 29th day, rats in phlorizin prevention groups, GS, and DC were treated by STZ injection. The symptoms recorded daily. The body weight and metabolic index were observed weekly for 35 days. Blood samples were collected from the posterior orbital venous plexus of the rats on days 31 and 35; then, FBG and serum lipid parameters were determined.

Finally in the two experiments, all the rats were sacrificed under ether anesthesia. All organs were visually inspected. Pancreas and liver samples were separated. Pancreatic cells and lipid accumulation in the liver were examined by HE and Oil Red O staining, respectively. The pathological changes were observed under light microscopy.

### 2.1 Statistical analysis

Statistical analyses were done with Statistica 7.0 software. All data were expressed as means ± *SD* and analyzed using analysis of variance (ANOVA followed by Tukey's test) for multiple comparisons. *p*‐value < .05 was considered to be statistically significant.

## 3 RESULTS

### 3.1 The manifestations of rats

**FIGURE 1:**
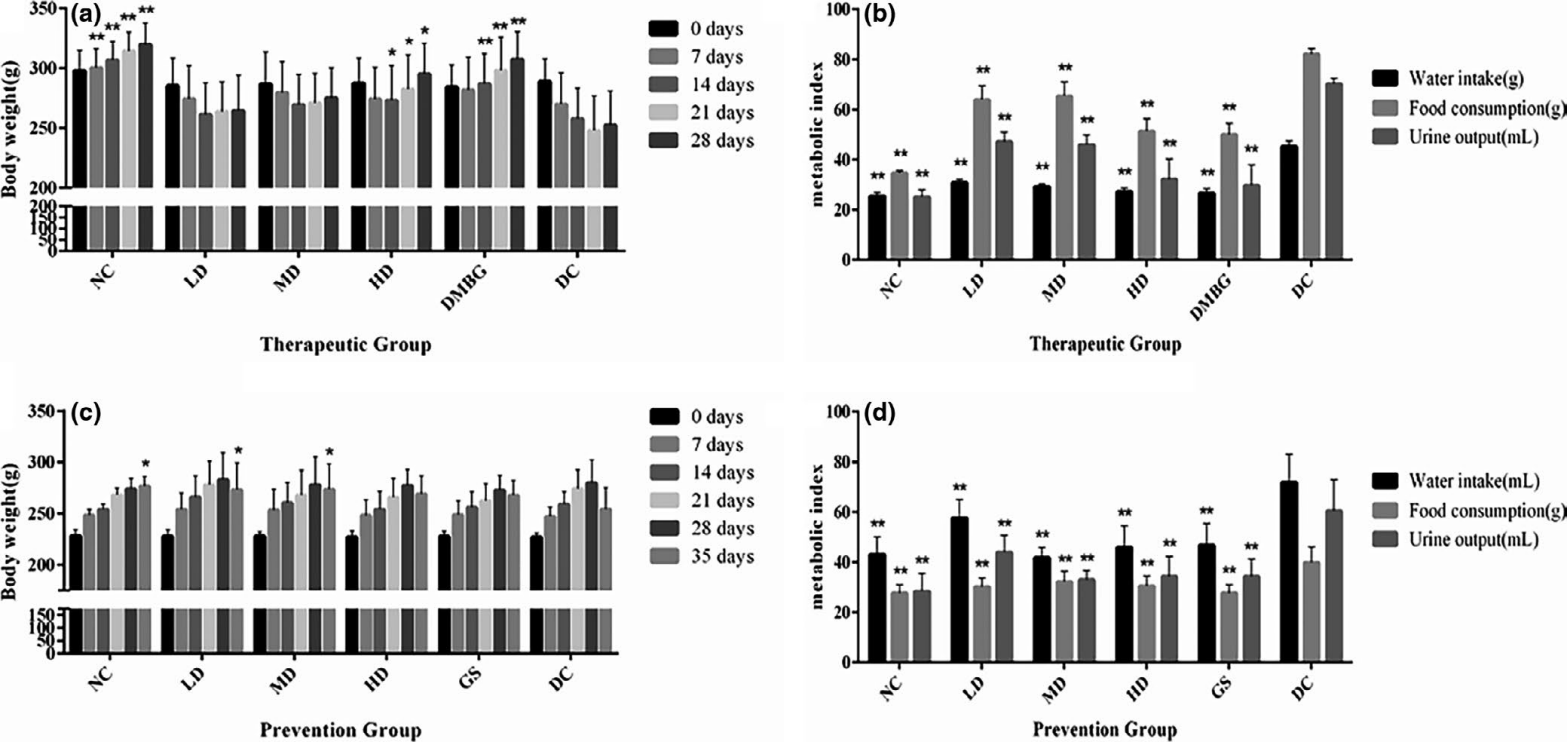
Effects of phlorizin on body weight of diabetic model rats. Body weight of therapeutic group (a), metabolic index in therapeutic group (b), body weight of prevention group (c), and metabolic index in prevention group (d), compared with DC on the same day or the same index. *p < .05, **p < .01

In therapeutic and preventive experiments, the manifestations of DC rats were spiritlessness, irregular and yellowish hair coat, increased food consumption and drinking volume, pungent smell, and damp of the bedding materials compared with NC rats after STZ injection. Symptoms improved including normal response, smooth and lustrous hair, and decreased food consumption, drinking volume, and urine output were observed in other groups (phlorizin, DMBG, and GS; Figure 1b,d).

In different groups, rat body weight (Figure 1a,c) was also observed. Both in phlorizin therapeutic and prevention experiments, body weight in DC rats was significantly lower in contrast with NC on 7 days after STZ injection (*p* < .01). During the therapy period, both phlorizin HD and DMBG effectively inhibited the weight loss of rats (*p* < .05), while phlorizin LD and MD were slightly inhibited the weight loss of rats (*p* > .05). During the prevention period, phlorizin LD and MD inhibited the weight loss (*p* < .05), whereas the effects of phlorizin HD and GS were not statistically significant (*p* > .05).

Body weight of therapeutic group (a), metabolic index in therapeutic group (b), body weight of prevention group (c), and metabolic index in prevention group (d) are compared with DC on the same day or the same index (*p < .05, **p < .01).

**FIGURE 2:**
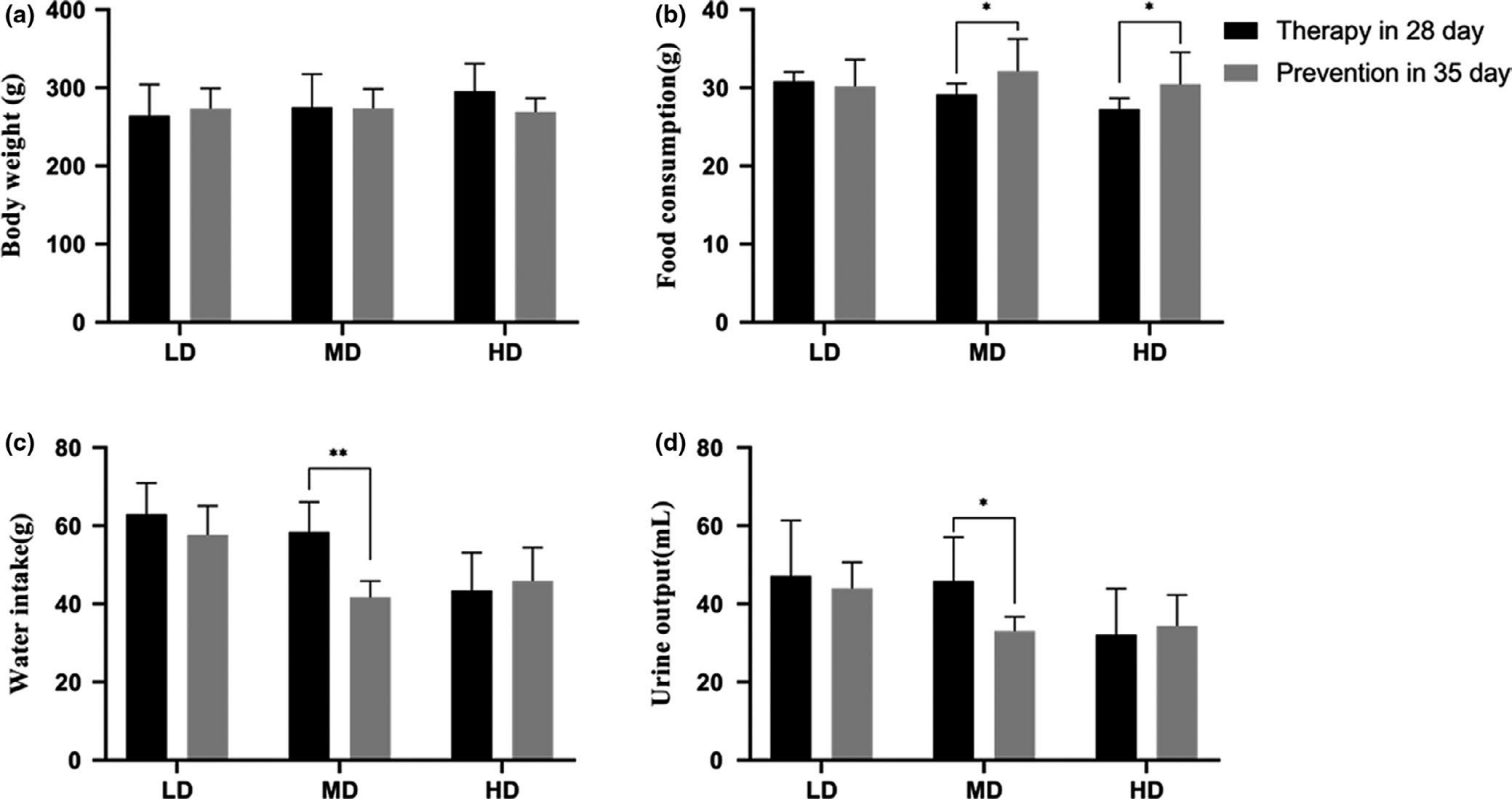
Effects of phlorizin at 35th day in prevention compared with the 28th day in therapeutic experiment. Body weight (a), food consumption (b), water intake (c), and urine output (d), 35th day in prevention compared with the 28th day in therapeutic experiment on the same index. *p < .05, **p < .01

At the end the of preventive period, phlorizin MD effectively improved (*p* < .05) the water intake and urine output compared with the 28th day in therapeutic experiment’ (Figure 2c,d).

Body weight (a), food consumption (b), water intake (c), and urine output (d), 35th day in prevention are compared with the 28th day in therapeutic experiment on the same index (*p < .05, **p < .01).

### 3.2 FBG levels of rats in each group

Fasting blood glucose levels in DC, phlorizin groups, DMBG, and GS were increased extremely significantly compared with NC (*p* < .01) at 3 days after STZ injection, showing diabetic model was made successfully.

**FIGURE 3:**
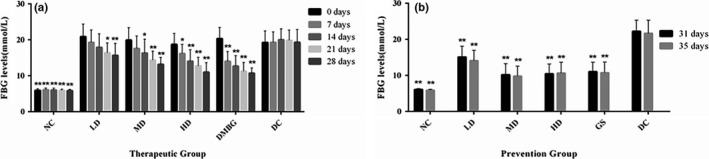
Effect of phlorizin on FBG levels. FBG levels of therapeutic groups (a) and FBG levels of prevention groups (b), compared with DC. *p < .05, **p < .01

In therapeutic test, compared with DC, FBG levels of phlorizin HD began to show significant difference on 7 days (*p* < .05), while significant differences existed in phlorizin LD, MD, and HD on 14, 21 and 28 days (*p* < .05 or *p* < .01; Figure 3a). In preventive experiment, there were extremely significant differences between DC and phlorizin preventive groups on 31 and 35 days (*p* < .01; Figure 3b).

Fasting blood glucose levels of therapeutic groups (a) and FBG levels of prevention groups (b) are compared with DC (*p < .05, **p < .01).

**FIGURE 4:**
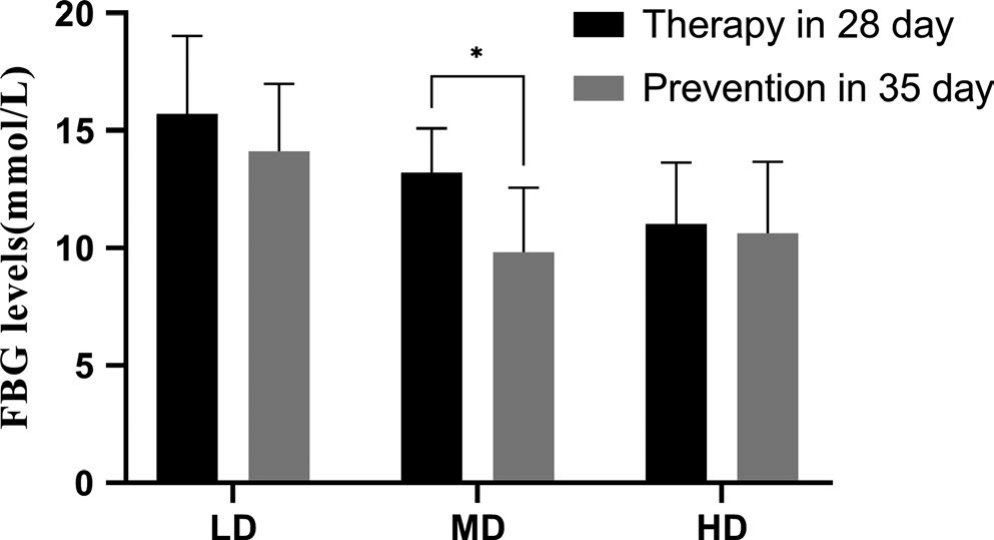
FBG levels of 35th day in prevention compared with the 28th day in therapeutic experiment. *p < .05

At the end of preventive period, phlorizin MD effectively decreased (*p* < .05) the FBG compared with the 28th day in therapeutic experiment (Figure 4).

Fasting blood glucose levels of 35th day in prevention are compared with the 28th day in therapeutic experiment (*p < .05).

### 3.3 Serum lipid levels in each group

**FIGURE 5:**
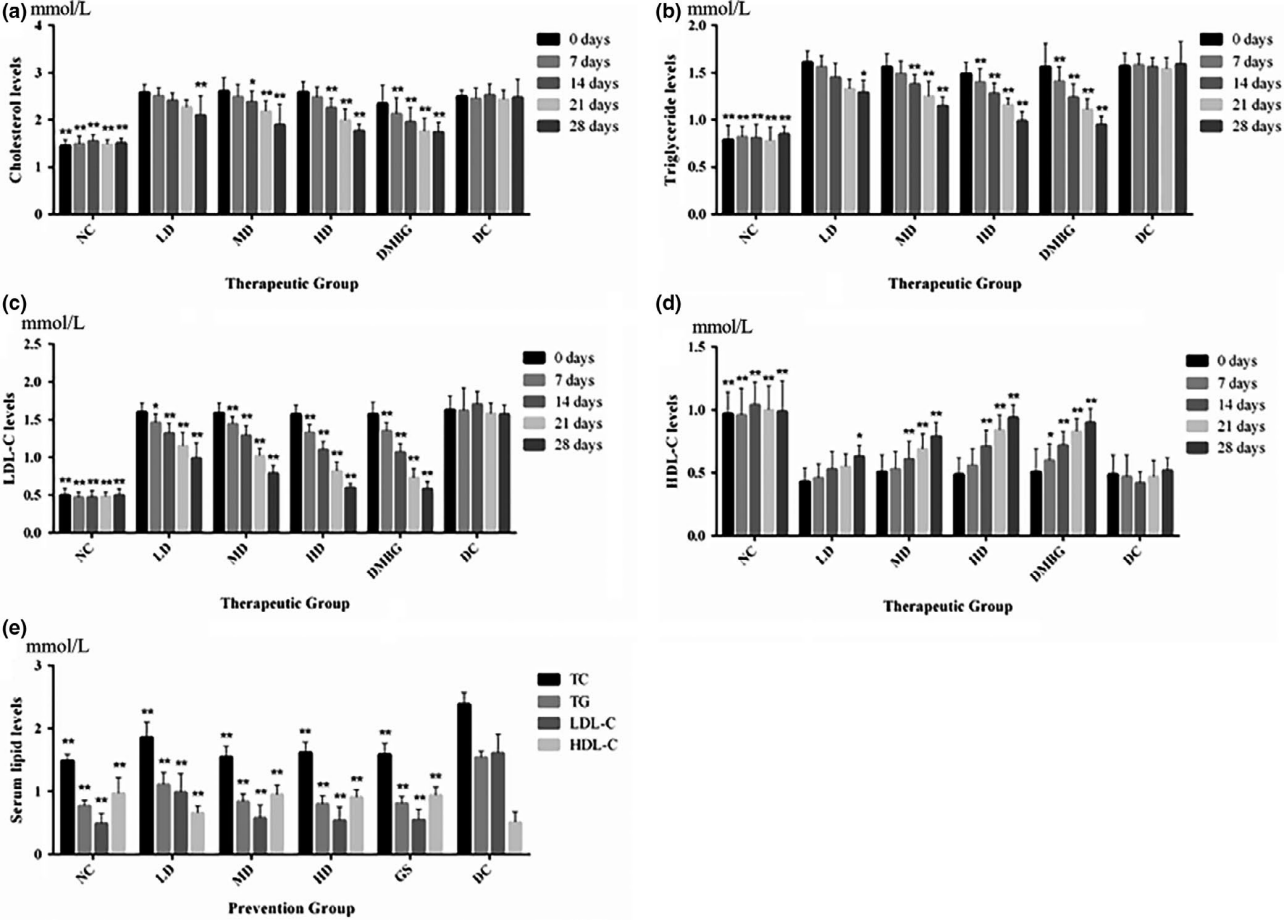
Effects of phlorizin on blood lipid levels in rats. TC levels of therapeutic group (a), TG levels of therapeutic group (b), LDL‐C levels of therapeutic group (c), HDL‐C levels of therapeutic group (d), and serum lipid levels of prevention group (e), compared with DC. *p < .05, **p < .01

Compared with NC, DC rats characterized by a remarkable increase in TC, TG, and LDL‐C levels, and extremely significant decrease in HDL‐C levels (*p* < .01), which indicated that alterations in serum lipid profile were closely related to the development of diabetes. For therapeutic groups, these levels of rats in phlorizin MD and HD had significant changes as compared with DC (*p* < .05 or *p* < .01), and the effects of phlorizin on diabetic rats displayed the dose threshold effect (Figure 5a–d). However, for preventive groups, serum lipid levels of rats treated with phlorizin were approximately equal to normal levels, but extremely significant difference to DC (*p* < .01; Figure 5e).

TC levels of therapeutic group (a), TG levels of therapeutic group (b), LDL‐C levels of therapeutic group (c), HDL‐C levels of therapeutic group (d), and serum lipid levels of prevention group (e) are compared with DC (*p < .05, **p < .01).

**FIGURE 6:**
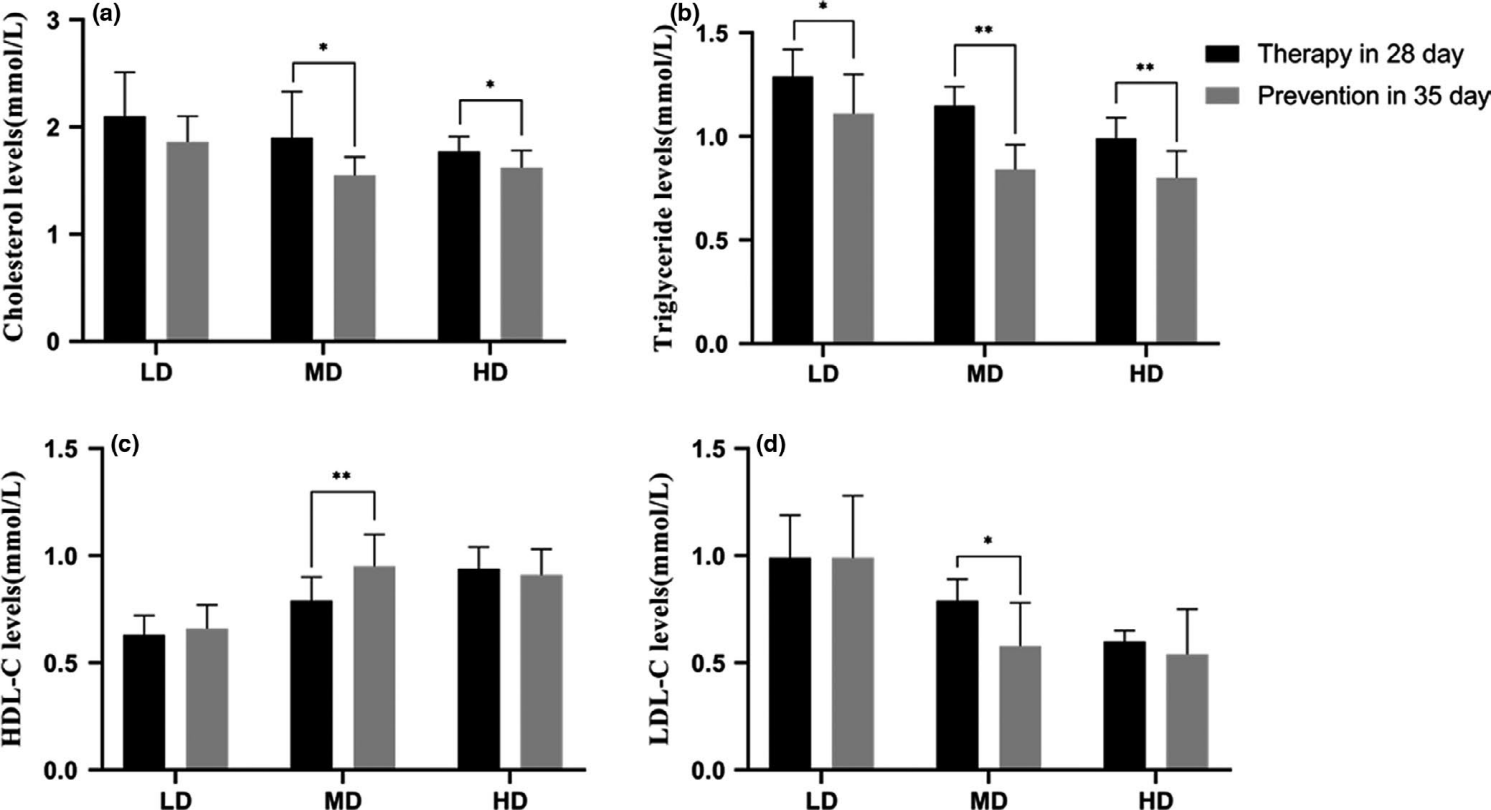
Effects of phlorizin on blood lipid levels at 35th day in prevention compared with at 28th day in therapeutic experiment. TC levels (a), TG levels (b), LDL‐C levels (c), and HDL‐C levels (d), 35th day in prevention compared with 28th day in therapeutic experiment on the same index. *p < .05, **p < .01

At the end of preventive period, phlorizin MD extremely decreased (*p* < .05) TC, TG, and LDL‐C levels (Figure 6a,b,d), and increased HDL‐C level compared with the 28th day in therapeutic experiment (Figure 6c).

TC levels (a), TG levels (b), LDL‐C levels (c), and HDL‐C levels (d) at 35th day in prevention compared with the 28th day in therapeutic experiment on the same index (*p < .05, **p < .01).

### 3.4 Effect of phlorizin on histopathological changes in diabetic rats

**FIGURE 7:**
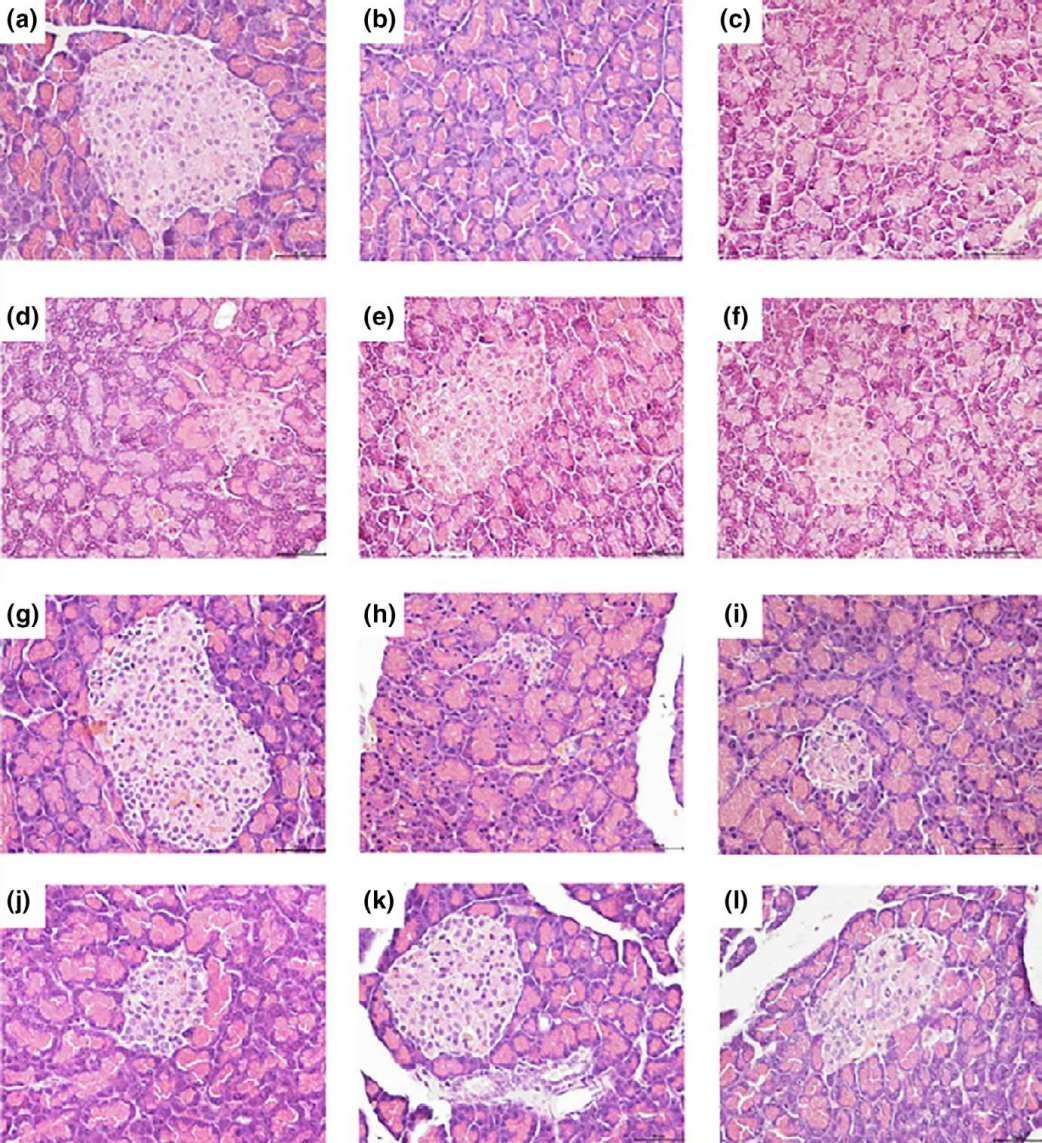
Histopathological analyses of pancreas taken from diabetic rats (HE 400×). NC (a), DC (b), LD (c), MD (d), HD (e), and DMBG in therapeutic experiment (f); NC (g); DC (h); LD (i); MD (j); HD (k); and GS in prevention group (l)

**FIGURE 8:**
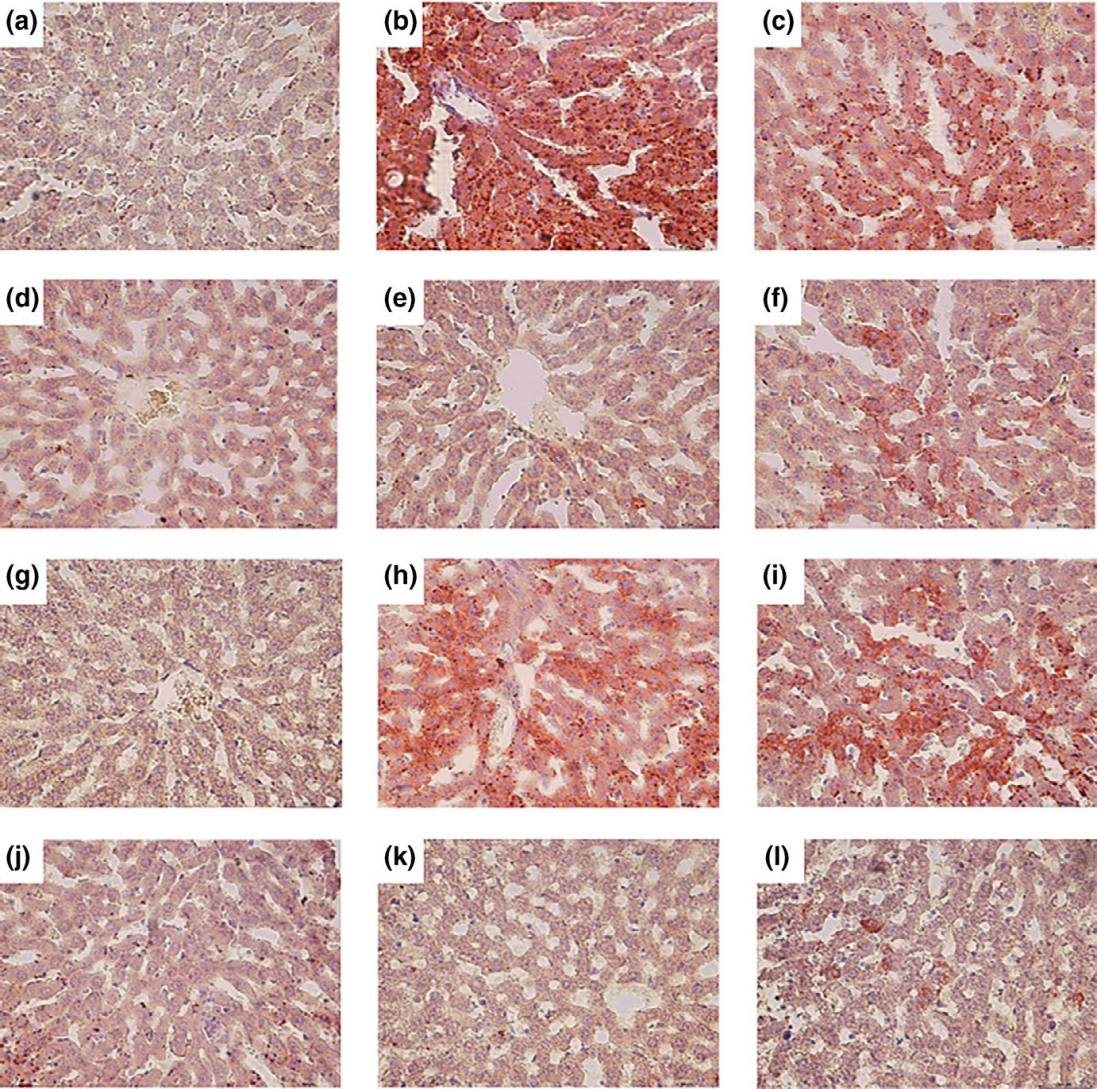
Histopathological analyses of liver taken from diabetic rats (HE 400×). NC (a), DC (b), LD (c), MD (d), HD (e), and DMBG in therapeutic experiment (f); NC (g); DC (h); LD (i); MD (j); HD (k); and GS in prevention group (l)

Histological observations revealed that pancreas presented smaller size, fewer, considerably smaller, and ill‐defined margins, and cellular decrease and degeneration of pancreas islets in DC rats (Figure 7b,h); meanwhile, Oil Red O staining demonstrated more lipid depositions were detected in liver cells in DC compared with NC (Figure 8a,g). Phlorizin and melbine could restore pathological damages of the pancreas in diabetic rats after therapy treatment (Figure 7b). Notably, these rats treated with 120 mg/kg phlorizin (Figure 7e) showed more number of islets and islet cells, and the shape and structure of islet cells remain normal. Rats treated with 30 mg/kg phlorizin (Figure 8c) exhibited more fat droplet were present in the liver cells, whereas the rats treated with 60 and 120 mg/kg phlorizin (Figure 8d,e) showed evidently decreased fat deposition. In preventive experiment, phlorizin seemed to protect against damaged pancreas islet and islet cells, especially 120 mg/kg phlorizin (Figure 7k) exerting the best effect.

## 4 DISCUSSION

Diabetes is a chronic disorder of metabolic disorders owing to islet cell dysfunction, insulin resistance, or both. The symptoms of diabetes include improving blood glucose, polydipsia, polyuria, polyphagia, and loss of body weight (Makena et al., 2018), the body weight loss indicates protein and fat catabolism owing to altered polyuria is caused by osmotic diuresis due to hyperglycemia in glucose homeostasis, and diabetic rats are treated with high‐fat diet (HFD)/STZ. The administration of HFD and STZ is the most common method for reproducing diabetes. HFD would cause insulin resistance, and STZ, a most common diabetogenic agent, is particularly toxic to islet β‐cells, reducing the release of insulin and then elevating the blood glucose level. This method has been widely used for preparation of diabetes and related therapeutic studies (Gilbert et al., 2011). In the present work, diabetic rat model was induced by HFD and intraperitoneal STZ (40 mg/kg). As expected, the diabetic model rats produced symptoms, such as spiritlessness, polyphagia, polydipsia, polyuria, and weight loss, increased fasting blood glucose (FBG) levels, cholesterol (TC), triglyceride (TG), low‐density lipoprotein cholesterol (LDL‐C) levels, and decreased high‐density lipoprotein cholesterol (HDL‐C) levels, pancreatic lesions, and liver steatosis. The present findings suggest phlorizin improved the symptoms of diabetic rats, but the rats in the high‐dose group lost weight and previous study (Zhou et al., 2013) have shown that sweet tea leaf extract could significantly decrease the levels of serum lipids, attenuate body weight gain and lower circulating leptin and insulin levels, reduce circulating CRP and resistin levels, and depress the expression of PPARγ and C/EBPα in epididymal adipose tissue of obese rats. Phlorizin can effectively reduce body weight, redress disturbance of glucose and lipid metabolism, and educe dangerous factors of obesity; it may play a role by downregulating the level of mTOR and its phosphorylation, up‐regulating GβL, Raptor expression, improving glucose, and lipid metabolism in liver tissue (Guo, 2013).

Hyperglycemia and dyslipidemia are common in human diabetes. The products that have therapeutic effects on hyperglycemia and hyperlipidemia would have a broad prospect for treating diabetes. Previous studies showed that phlorizin inhibited the reabsorption of sodium glucose cotransporter (SGLT) and reduces glucose re‐entry into the systemic circulation (Mudaliar et al., 2015). Phlorizin not only reduces the blood glucose concentration in diabetic rats, but also restores the function of β‐cells and insulin sensitization (Rossetti et al., 1987). Zhao et al. (2004) found that treatment with phlorizin reduced blood glucose and increased glucose in urine, confirming the hypoglycemic effect of phlorizin. The results obtained in the present study were consistent with the previous reports, which supported the opinion that phlorizin had effectively reduced high blood glucose and improved lipid metabolism in the model of experimental diabetes. Both in the therapeutic and preventive experiment for diabetic model rats, treatment with phlorizin was effective in the improvement of typical symptoms of diabetes such as spiritlessness, polyphagia, polydipsia, polyuria, and hyperglycemia in diabetic model rats, as well as significantly reduced the improved FBG levels in diabetic model rats. Hypoglycemic effect of phlorizin could be associated with histone acetylation, glucose homeostasis, the acetylcholine catabolic process, the pentose catabolic process, purine nucleoside transport, anion transport, and calcium‐ion homeostasis (Ran et al., 2019). This suggests that the significant weight gain in diabetic rats was related to phlorizin.

Diabetes tends to increase LDL‐C and decrease HDL‐C levels in serum, triggering coronary occlusions and blocks. So, it is essential to control not only blood glucose levels but also blood lipid levels (Rangika et al., 2015). Until recently, numerous studies have demonstrated phlorizin has a potent effect on lowering blood lipid (Dong et al., 2006). Some studies (Cai et al., 2013) have shown that phlorizin can prevent diabetic cardiomyopathy in diabetic mice and greatly reduce the level of blood lipid in the mice. In the present study, regardless of prevention or treatment experiment, phlorizin exhibited the beneficial effects on regulating the levels of serum lipid in diabetic rats, including significant decrease in TC, TG, and LDL‐C levels, and significant increase in HDL‐C level. However, the effect showed a time gap between different dose continuous treatment which revealed the “threshold” of dosage and needs time to accumulate under the experimental dose. At the end of the two experimental periods, prophylactic administration showed more lower FBG, TC, TG, and LDL‐C and higher HDL‐C than therapeutic, especially in MD administration (all groups have been continuous administrated phlorizin for 28 days), which revealed the preventive treatment can effectively control the occurrence of STZ‐induced diabetes and reduce its severity, and also can be attributed to the threshold dose. In other words, the minimum efficacy dose for anti‐STZ‐induced diabetes just obtained during the therapeutic period; however, it has been attained by accumulation before animal model making (STZ injected) in preventive experiment. Moreover, HD administrated shortened the accumulation and LD extended the process, so there only was slightly different in the comparison by HD or LD treatment, respectively. The study can be a support for prophylactic use of phlorizin and commonly intake of *L. polystachyus* Rehd for diabetes.

The previous study had revealed that administration with ST extract (75, 150, and 300 mg/kg) noticeably reduced the C/EBPa and PPARg gene expression levels in diet‐induced obese rats (Zhou et al., 2013). Hence, phlorizin may directly influence various lipid regulation systems.

Improvement in pancreas and liver histology in phlorizin HD group indicated that phlorizin might exhibit protective effects in pancreatic islet and islet cells, increased insulin secretion, restore pancreatic β‐cells function and blood glucose level, and reduce lipid aggregation in hepatic tissue in diabetic rats.

## 5 CONCLUSION

In conclusion, phlorizin from *L. polystachyus* Rehd exerts therapeutic and preventive effects on hyperglycemia and hyperlipidemia in HFD‐STZ‐induced diabetic rats by the following ways: (a) improvement of typical syndromes of diabetes including polyphagia, polydipsia, polyuria, and weight loss, (b) decreasing FBG level, and serum TC, TG, and LDL‐C levels, and increasing HDL‐C level, (c) restoring the structure of damaged islets of pancreases and islet cells, and decreasing fat deposition in hepatic cells. This study has confirmed that phlorizin from sweet tea has the therapeutic and preventive effects on hyperlipidemia and hyperglycemia in diabetes rats. Phlorizin can be used as a natural health drink to prevent diabetes.

## CONFLICT OF INTEREST

The authors declare no conflict of interest.

## Data Availability

The data that support the findings of this study are available on request from the corresponding author. The data are not publicly available due to privacy or ethical restrictions.
